# If Artificial In Vitro Microenvironment Can Influence Tumor Drug Resistance Network via Modulation of lncRNA Expression?—Comparative Analysis of Glioblastoma-Derived Cell Culture Models and Initial Tumors In Vivo

**DOI:** 10.1007/s10571-020-00991-3

**Published:** 2020-11-27

**Authors:** Monika Witusik-Perkowska, Dariusz J. Jaskólski, Paweł P. Liberski, Janusz Szemraj

**Affiliations:** 1 Department of Medical Biochemistry, Medical University of Lodz, 6/8 Mazowiecka Str, 92-215 Lodz, Poland; 2 Department of Neurosurgery and Neurooncology, Medical University of Lodz, Barlicki University Hospital, Lodz, Poland; 3 Department of Molecular Pathology and Neuropathology, Medical University of Lodz, Lodz, Poland

**Keywords:** lncRNA, Glioblastoma, Cell culture, Tumor drug resistance

## Abstract

The tumor resistance of glioblastoma cells in vivo is thought to be enhanced by their heterogeneity and plasticity, which are extremely difficult to curb in vitro. The external microenvironment shapes the molecular profile of tumor culture models, thus influencing potential therapy response. Our study examines the expression profile of selected lncRNAs involved in tumor resistance network in three different glioblastoma-derived models commonly utilized for testing drug response in vitro. Differential expression analysis revealed significant divergence in lncRNA profile between parental tumors and tumor-derived cell cultures in vitro, including the following particles: MALAT1, CASC2, H19, TUSC7, XIST, RP11-838N2.4, DLX6-AS1, GLIDR, MIR210HG, SOX2-OT. The examined lncRNAs influence the phenomenon of tumor resistance via their downstream target genes through a variety of processes: multi-drug resistance, epithelial–mesenchymal transition, autophagy, cell proliferation and viability, and DNA repair. A comparison of in vivo and in vitro expression identified differences in the levels of potential lncRNA targets, with the highest discrepancies detected for the *MDR1*, *LRP1*, *BCRP* and *MRP1* genes. Co-expression analyses confirmed the following interrelations: MALAT1–TYMS, MALAT1–MRP5, H19–ZEB1, CASC2–VIM, CASC2–N-CAD; they additionally suggest the possibility of MALAT1–BCRP, MALAT1–mTOR and TUSC7–PTEN interconnections in glioblastoma. Although our results clearly demonstrate that the artificial ex vivo microenvironment changes the profile of lncRNAs related to tumor resistance, it is difficult to anticipate the final phenotypic effect, since this phenomenon is a complex one that involves a network of molecular interactions underlying a variety of cellular processes.

**Electronic supplementary material:**

The online version of this article (10.1007/s10571-020-00991-3) contains supplementary material, which is available to authorized users.

## Introduction

Glioblastoma (GB) is the most common, aggressive and practically incurable brain tumor (Alexander and Cloughesy 2017). GB cells demonstrate considerable molecular and phenotypic heterogeneity, indicating the complexity of the mechanisms underlying their drug-resistant phenotype and highlighting the need for multidirectional research to identify novel therapeutic modalities (Parker et al. 2016; Cai and Sughrue 2017; Akgül et al. 2019). Apart from the crucial pathways recognized as factors underlying the phenomenon of GB aggressiveness and resistance, a number of recent investigations have examined the role of non-coding RNA in relation to GB pathogenesis, the bases of an incurable phenotype and the possibility of creating some novel therapeutic modalities (Siddharth et al. 2015; Li et al. 2018). Non-coding RNAs (miRNAs, lcnRNAs) have the capability to influence a variety of cellular processes underlying the phenomenon of tumorigenesis and therapy resistance, such as proliferation, migration, invasiveness, angiogenesis induction, apoptosis regulation, autophagy, stemness state and differentiation potential, as well as EMT (epithelial-mesenchymal transition) status, chemo- and radiosensitivity (Rynkeviciene et al. 2018; Heery et al. 2017).

Tumor-derived cell cultures represent a common model for the study of mechanisms of drug resistance or the search for new therapeutic approaches. However, because artificial in vitro conditions may influence the genotype and phenotype of neoplastic cells, including their potential response to treatment, a number of recent studies have examined the selection of experimental tumor cell culture models (Balvers et al. 2017; Ledur et al. 2017; Robertson et al. 2019; Caragher et al. 2019; da Hora et al. 2019).

Extrinsic in vitro factors are known to influence the molecular background of GB drug resistance and consequently the efficiency of treatment, as well as the mechanisms/pathways of cell death (Witusik-Perkowska et al. 2017). Additionally, it has been confirmed that the artificial in vitro microenvironment changes the profile of miRNAs related to glioblastoma resistance (Witusik-Perkowska et al 2019).

Recent papers emphasize the role of events engaging lncRNAs in the phenomenon of tumor therapy sensitivity (Heery et al. 2017; Zhao et al. 2019; Wang et al. 2020). As such epigenetic mechanisms, including non-coding RNA profile, can be easily influenced by environmental factors, these should be taken into account when determining the utility or representativeness of artificial in vitro models. Therefore, our study is aimed at exploring potential influence of artificial ex vivo microenvironment on the expression profile of lncRNAs related to GB drug resistance, since it has not been reported yet.

The literature survey was performed to identify lncRNAs that may be engaged in glioblastoma therapy resistance, particularly those related to temozolomide (TMZ) response, the drug used as standard in GB treatment (Zhang and Leung 2014; Peng et al. 2018; Strobel et al. 2019).

The current study examines the potential influence of ex vivo conditions on the profile of selected lncRNAs (MALAT1, CASC2, H19, TUSC7, XIST, RP11-838N2.4, DLX6-AS1, GLIDR, MIR210HG, SOX2-OT) interconnected with GB resistance molecular network, including their potential target genes.

Such an approach allows for assessing the utility of the different GB in vitro models as tools to investigate tumor drug resistance in context of lncRNA profile. Additionally, it enables to evaluate, if change in lncRNA level induced by ex vivo factors, influences an expression of interrelated target genes.

## Materials and Methods

### Glioblastoma Cell Cultures

Glioblastoma cell cultures were derived from tumor samples obtained from the Department of Neurosurgery and Neurooncology, Medical University of Lodz, Poland. All procedures (i.e. experiments with human tumor-derived cells) were performed in accordance with the ethical standards of the Bioethics Committee of the Medical University of Lodz (reference RNN/148/08/KE and RNN/160/15/KE). Glioblastoma cultures were derived from four tumors classified according to WHO criteria as Glioblastoma, NOS—not otherwise specified; (i.e. the genetic status of *IDH*—isocitrate dehydrogenase was not verified) (Louis et al. 2016).

The tumor sample was processed, and its cells cultured, according to previously published protocol (Witusik-Perkowska et al. 2019). Briefly, the cells derived from initial tumors were cultured under three different conditions: adherent culture in serum-supplemented medium (DMEM/F12 with 10% fetal bovine serum (FBS)), adherent culture in serum-free conditions on commercially available vitronectin-mimicking synthetic peptide-acrylate plates (neurobasal medium—NBM with G5, NSC) (Corning R SynthemaxTMSurface) and spheroid culture in serum-free conditions (NBM medium with N2, B27, epidermal growth factor—EGF, basic fibroblast growth factor—bFGF and heparin). The products used for cell culture generation and growing (DMEM-F/12, growth factors and supplements) were purchased from Thermo Fisher Scientific, including commercially available Gibco™ Fetal Bovine Serum (US). Further analyses were performed with the use of cells cultured under particular conditions for at least two to three passages.

To eliminate the risk of overgrowth of non-tumoral cells in vitro, the previously established protocol of culture status monitoring was applied (Witusik-Perkowska et al. 2019). The presence of neoplastic cells in the cultures was verified by immunofluorescence detection of astrocytoma-associated antigens (AAAs) IL13Rα2, Fra-1 and EphA2 and confirmed at DNA level, e.g. by loss of heterozygosity (LOH) analyses. The immunofluorescence method and LOH analysis technique were performed as described previously (Witusik-Perkowska et al. 2014, 2019).

### Total RNA Isolation and cDNA Synthesis

The initial tumors and the cells cultured in different models for at least two or three passages were subjected to total RNA isolation using the Total RNA Mini Plus kit (A&A Biotechnology; Poland). The samples were treated with DNaseI (Sigma-Aldrich) and cDNA synthesis was performed with the SensiFAST™ cDNA Synthesis Kit according to the manufacturer’s instructions (Bioline, UK).

### lncRNA Expression Profiling via Quantitative Real-Time RT-PCR

lncRNA expression was determined using a single primer assay based on real-time PCR. Pre-designed commercially available RT2 lncRNA qPCR Assays were used: *MALAT1*, LPH18065A; *CASC2*, LPH01409A; *H19,* LPH01147A; *TUSC7,* LPH15183A; *XIST,* LPH08103A; *RP11-838N2.4,* LPH25144A; GLIDR, LPH00981A; *MIR210HG,* LPH15919A; *SOX2-OT*, LPH15037A; (Qiagen).

Real-time PCR analysis was performed using the QuantiTect SYBR® Green PCR Master Mix Kit according to the manufacturer’s instructions. The analyzed lncRNA expression was normalized using *GUSB* (β-glucuronidase) gene. Each sample was amplified in a reaction volume of 20 μl, containing cDNA, QuantiTect SYBR® Green PCR Master Mix and an appropriate primer assay. Real-time PCR was performed using a Stratagene Mx3005P instrument (Agilent). The results were analyzed using Stratagene Mx3005P software. The cycling conditions were set according to the manufacturer’s protocol. To confirm the specificity of the amplification, the gene dissociation curve was considered in each case. Normalized relative expression levels of the examined lncRNAs in the tested samples were calculated against a control value according to the modified 2ΔΔCT method, based on the mean CT value of the sample (Livak and Schmittgen 2001; Wilhelm and Pingoud 2003).

$$\begin{aligned} &\Delta \Delta {\text{CT}} = \Delta {\text{CT}}\left( {\text{target sample}} \right) - \Delta {\text{CT}}\left( {\text{control sample}} \right) = \left( {{\text{CTref}}_{{{\text{tar}}}} - {\text{CTlncRNA}}_{{{\text{tar}}}} } \right) - \left( {{\text{CTref}}_{{{\text{cont}}}} - {\text{CTlncRNA}}_{{{\text{cont}}}} } \right) \hfill \\ &{\text{lncRNA fold change relative to control}} = { 2}^{{\Delta \Delta {\text{CT}}}} \hfill \\ \end{aligned}$$

To evaluate the relative expression in target samples, commercially available RNA from a human brain (Total RNA, Brain, Human; Agilent Technologies) or RNA samples derived from initial tumors were used as controls when examining expression in corresponding culture models.

### Expression of the Selected Target Genes via Quantitative Real-Time RT-PCR

Expression of the selected target genes at mRNA level was assessed with the use of a single primer assay based on real-time PCR. The primer sequence are provided as Table S1. Real-time PCR analysis was performed using the QuantiTect SYBR® Green PCR Master Mix Kit according to the manufacturer’s instructions. *GUSB* was used as reference to normalize the analyzed gene expression. Each sample was amplified in a reaction volume of 20 μl, containing cDNA, QuantiTect SYBR® Green PCR Master Mix and appropriate primer assay. Real-time PCR was performed using a Stratagene Mx3005P instrument (Agilent). The results were analyzed using Stratagene Mx3005P software. To confirm the specificity of the amplification, the gene dissociation curve was considered in each case. Normalized relative expression levels of the examined genes in the tested samples (ex_tar_) were calculated against a control value according to the modified 2ΔΔCT method, based on the mean CT value of the sample.

$$\begin{aligned} &\Delta \Delta {\text{CT}} = \Delta {\text{CT}}\left( {\text{target sample}} \right) - \Delta {\text{CT}}\left( {\text{control sample}} \right) = \left( {{\text{CTref}}_{{{\text{tar}}}} - {\text{CTex}}_{{{\text{tar}}}} } \right) - \left( {{\text{CTref}}_{{{\text{cont}}}} - {\text{CTex}}_{{{\text{cont}}}} } \right) \hfill \\ &{\text{mRNA fold change relative to control}} = {2}^{{\Delta \Delta {\text{CT}}}} \hfill \\ \end{aligned}$$

To evaluate the relative expression in target samples, commercially available RNA from a human brain (Total RNA, Brain, Human; Agilent Technologies) or RNA samples derived from initial tumors were used as controls when examining expression in corresponding culture models.

### Statistical and Computational Analysis

The expression of examined lncRNAs in GBs was compared to those in normal control samples based on TCGA and GTEx datasets using GEPIA2 (Gene Expression Profiling Interactive Analysis) web software: http://gepia2.cancer-pku.cn/#index (Tang et al. 2019a, b).

The experimental data for further analysis yielded from at least three replicates (n = 3).

Heat maps and clustering analyses were generated from the means of ΔΔCT values (relative to control—human brain) with the use of Gitools software (Perez-Llamas et al. 2011). Negative ΔΔCT values were considered as gene underexpression in relation to control (HB, human brain), and positive ΔΔCT values were considered as gene overexpression.

The expression data (ΔΔCt values) were analyzed by non-parametric tests. The differences between more than two groups were first analyzed with the Kruskal–Wallis test. If this difference proved significant, individual groups were further investigated using the Conover-Inman non-parametric post hoc test. *p* < 0.05 was considered significant. The output of statistical analysis is provided as Table S3.

Finally, the results were exhibited as mean ΔΔCT values (expression relative to control HB) or as fold change in relation to appropriate parental tumors.

Co-expression analysis for our experimental data was performed by means of Spearman’s correlation analysis; the obtained results were presented as a correlation matrix for Spearman coefficient *r* and *p* value. Analyses were generated via Gitools software using ΔΔCT values (relative to control—human brain); *p* < 0.05 was considered significant.

The quantitative data used for heat map generation and correlation analysis for all lncRNAs and the target genes, expressed as ΔΔCT values relative to control (HB; human brain) are Supplemented as Table S2.

Correlation analysis was also performed with the use of GEPIA2 interactive web software based on existing datasets for GB tumors.

## Results

### Basic Characteristic of Glioblastoma-Derived Models In Vitro

Glioblastoma cells were derived from four tumors: G108, G113, G114, G116. Tumors were classified as glioblastoma according to WHO criteria, based on histopathological diagnosis; (representative results of immunohistochemistry had been published previously; Witusik-Perkowska et al. 2014). The general description of tumors including basic clinical data are summarized in Table 1. Experiments were designed to culture the cells in three different conditions: an adherent culture in serum-supplemented medium (10% adh); a spheroid serum-free culture (0% sph); and an original method of adherent culture on a synthetic vitronectin-mimicking surface in serum-free medium (0% adh), as described previously (Witusik-Perkowska et al. 2019). The G108, G114 and G116 tumors exhibited the ability to grow in all applied models, while the G113 tumor did not create stable spheroids. In comparison to our previous study, no changes in protocol of culture have been made. Despite this fact, the differences in ability to spheroid formation were noticed for G113 and G114 tumors. The observed divergence may have resulted from an inherent tumor cell plasticity and intra-heterogeneity influencing cell behavior (Davis et al. 2019; Fanelli et al. 2020).

**Table 1:** Basic clinical characteristics of tumors

Sample	Histopathology	Age	Sex	Radiotherapy/chemotherapy
G108	Glioblastoma	59	F	No
G113	Glioblastoma	73	F	No
G114	Glioblastoma	65	M	No
G116	Glioblastoma	79	F	No

Glioblastoma-derived cells in vitro demonstrated expression of IL13Rα2, EphA2 and Fra-1, described previously as AAAs (astrocytoma-associated antigens) facilitating tumor culture establishment (Witusik-Perkowska et al. 2014, 2019).

**Fig. 1:**
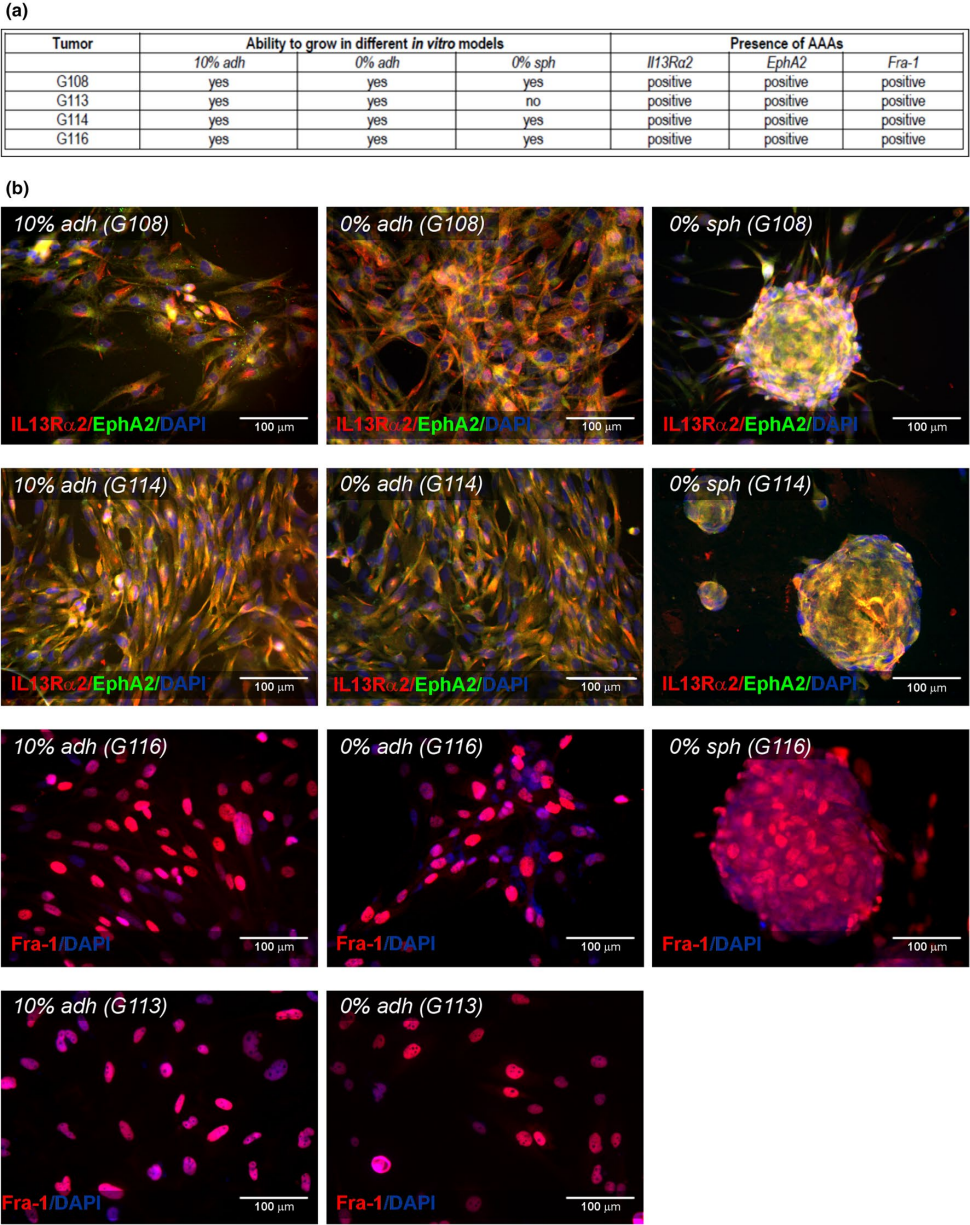
Basic characteristics of glioblastoma-derived models in vitro. The study included three different ex vivo GB models: adherent culture supplemented with 10% FBS (10% adh); serum-free adherent culture (0% adh) and serum-free spheroid culture (0% sph). G108-, G114- and G116- derived cells were able to grow in all culture conditions, while G113 did not create stable spheroids. The status of neoplastic cell presence in culture was verified with the use of AAAs: IL13Rα2, EphA2 and Fra-1. All GB-derived culture models manifested expression of examined markers (**a**). The representative microphotographs of immunofluorescence results revealing AAAs presence in GB-derived cells in vitro (**b**)

A summary of experimental design and basic cell culture characteristics is presented in Fig. 1.

### Differential Expression of lncRNAs Related to Tumor Resistance in GBs In Vivo and In Vitro

**Fig. 2:**
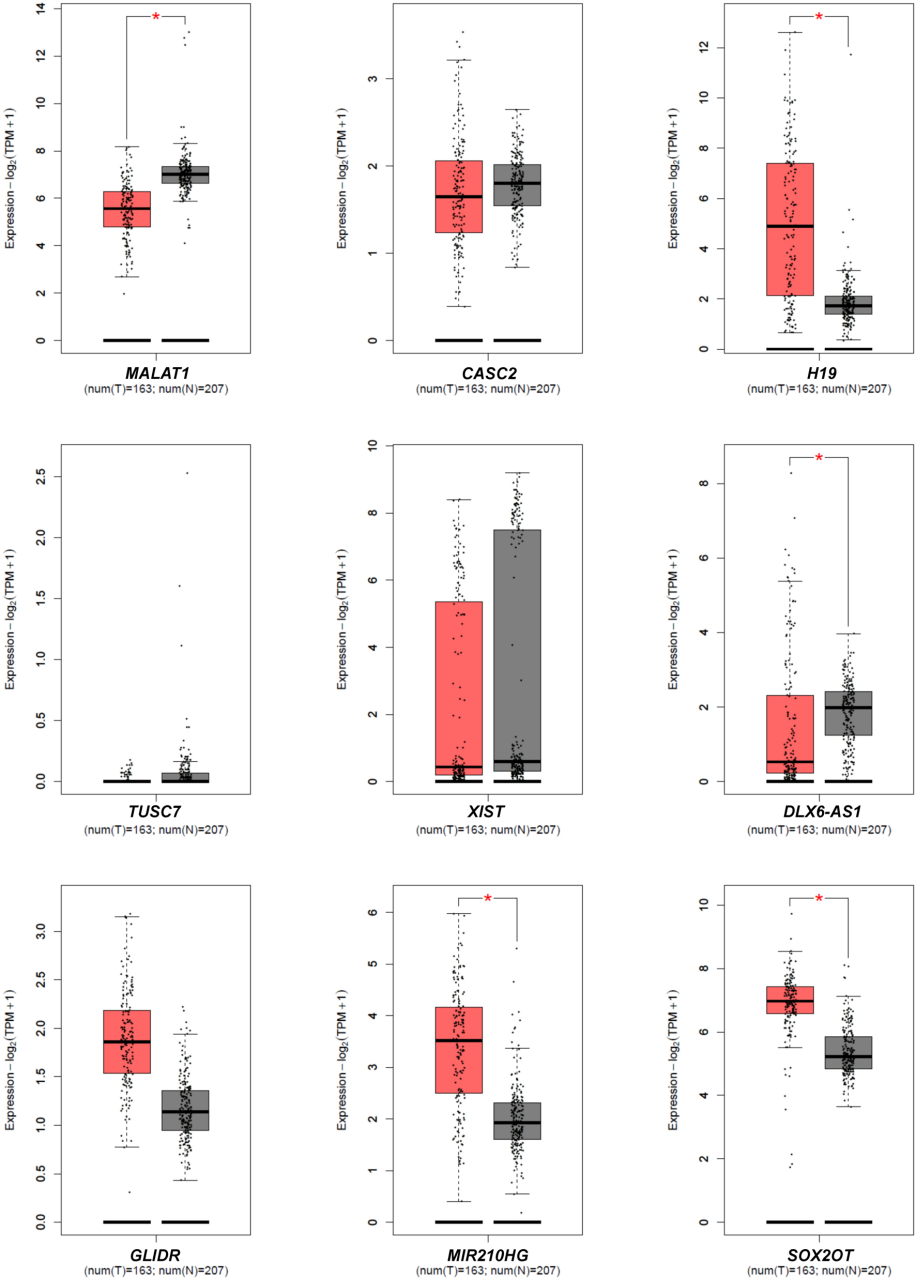
Validation of expression status of examined lncRNAs based on TCGA and GTEx data in glioblastoma. Box plots generated via GEPIA2 tool demonstrate results of differential analysis of the examined lncRNA levels using data from the TCGA and GTEx for GBs (T; *n* = 163) compared to normal control samples (N; *n* = 207); *p* < 0.05

The following lncRNAs engaged in glioblastoma therapy resistance were selected: MALAT1, CASC2, H19, TUSC7, XIST, RP11-838N2.4, DLX6-AS1, GLIDR, MIR210HG, SOX2-OT (Zhang and Leung 2014; Peng et al. 2018). The expression status of these lncRNAs in GB tumors was determined using GEPIA2 interactive software (Tang et al. 2019a, b*)*. This web-based tool allowed to perform a differential expression analysis using TCGA and GTEx data obtained for the 163 GBs and the 207 normal control samples yielding the results presented in Fig. 2. GEPIA2-derived analysis demonstrated aberrant expression of some lncRNAs in GB tumors. Additionally, the literature values emphasize the dependence of those lncRNAs on TMZ responsiveness (Table 2). The expression status of examined lncRNAs was also assessed in the GB initial tumors used in this study for cell culture generating. Our own findings demonstrating the expression of particular lncRNAs in the analyzed GB tumors (G108, G113, G114, G116) compared to control (HB) are included in Table 2.

**Table 2:** Expression of lncRNAs related to tumor drug resistance in GB tumors

lncRNA	Expression relative to HB (fold change to 1)				Expression status in GB according to GEPIA2 analysis	Expression status in GB according to literature
	*G108T*	*G113T*	*G114T*	*G116T*		
MALAT1	0.04 [0.015, 0.064]	0.28 [> 0.000, 0.6526]	0.17 [0.090, 0.249]	0.54 [> 0.000, 1.334]	Downregulated	Expression level dependent on TMZ responsiveness (Chen et al. 2017)
CASC2	0.05 [0.018, 0.081]	0.56 [0.019, 1.101]	0.33 [> 0.000, 0.727]	1.43 [> 0.000, 3.053]	Differences non-significant	Downregulated (Liao et al. 2017); Dependent on TMZ responsiveness (Jiang et al. 2018)
H19	0.01 [0.003, 0.016]	2.02 [0.540, 3.499]	15.5 [3.82, 27.18]	1.42 [> 0.000, 3.481]	Upregulated	Upregulated, correlates with TMZ resistance in glioma patients (Jiang et al.^2016^)
TUSC7	0.0013 [0.0011, 0.0014]	0.23 [0.0072, 0.4528]	0.51 [0.016, 1.003]	5.51 [> 0.000, 11.124]	Differences non-significant	Negatively correlated to TMZ resistance in GB (Shang et al. 2018)
XIST	0.15 [> 0.000, 0.398]	0.67 [0.371, 0.968]	0.0001 [> 0.000, 0.000216]	1.93 [> 0.000, 5.780]	Differences non-significant	Upregulated (Du et al. 2017; Yao et al. 2015)
RP11-838N2.4	0.08 [0.055, 0.104]	0.6 [> 0.000, 1.519]	0.91 [> 0.000, 2.723]	29.73 [> 0.000, 67.091]	No data	Correlated with glioma grading and risk of GB relapse (Liu et al. 2016)
DLX6-AS1	0.54 [> 0.000, 1.185]	0.06 [> 0.000, 0.123]	0.22 [0.076, 0.363]	1.45 [0.940, 1.959]	Downregulated	Upregulated (Li et al. 2019)
GLIDR	0.18 [0.052, 0.307]	1.99 [0.648, 3.331]	0.76 [> 0.000, 1.555]	1.65 [0.138, 3.161]	Differences non-significant	Downregulated (Zhang et al. 2013)
MIR210HG	0.44 [0.026, 0.853]	0.26 [> 0.000, 0.562]	4.95 [1.926, 7.973]	23.65 [> 0.000, 64.439]	Upregulated	Upregulated (Min et al. 2016)
SOX2-OT	0.13 [0.034, 0.225]	0.58 [0.118, 1.041]	0.22 [> 0.000, 0.458]	0.34 [0.005, 0.674]	Upregulated	Upregulated (Su et al. 2017)

Data exhibited as fold change [mean: 95%CI] in relation to expression level in control human brain; underexpression = fold change < 1; overexpression = fold change > 1

GEPIA2 analysis based on available datasets; comparison of expression data for the163 GB vs 207 normal controls

*HB* human brain, *GB* glioblastoma

**Fig. 3:**
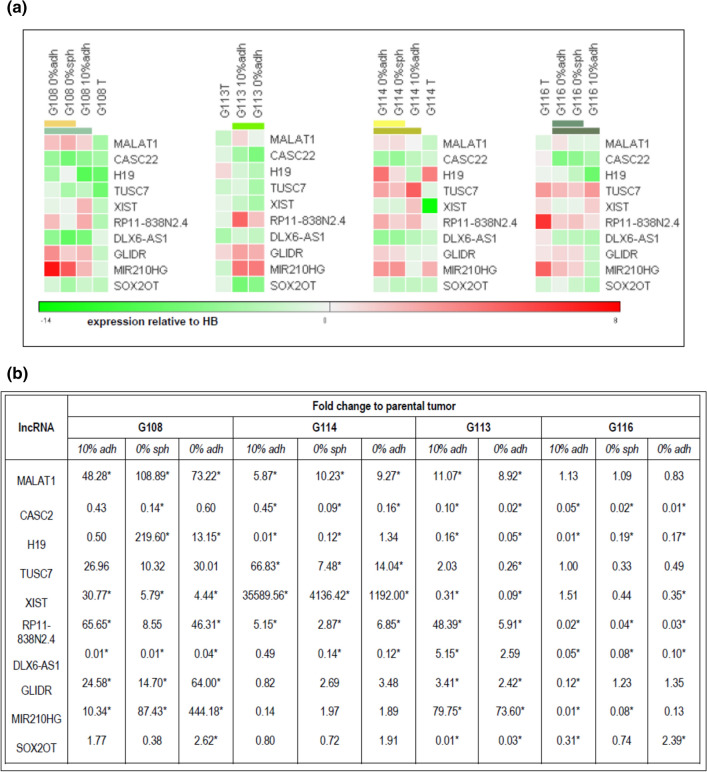
Results of expression analysis for the lncRNAs related to GB drug resistance. Heat maps were generated based on ΔΔCT values (relative to control—human brain; HB). Hierarchical clustering analyses highlighted divergence between lncRNAs profiles of initial tumors and the cells in vitro, and demonstrated closer similarity of serum-free models in comparison to serum-supplemented cultures. The results of clustering according to the samples (columns) are depicted as colored bars in the heat map representing the hierarchical tree splitting at different levels. The root of the tree is located at the bottom, the leaves at the top (**a**). To visualize the scale of lncRNA profile changes, the data were expressed as fold change of level in particular models relative to parental tumor. The comparative analysis confirmed the significance of differences between in vitro models and the tumors in vivo (*); *p* < 0.05

To compare the profiles of the selected lncRNAs in vivo and in the in vitro models, differential expression analysis was performed. The obtained data were presented as heat maps generated from mean ΔΔCt values (expression relative to HB), giving overall picture of examined lncRNA profile in particular GBs in vivo and in vitro. Hierarchical clustering analysis revealed a discrepancy in lncRNA expression profiles between initial tumors and in vitro glioblastoma cells for every analyzed tumor. Regarding the differences between particular in vitro models, the closest similarity was observed between the two serum-free models: 0%sph and 0% adh (Fig. 3a).

To better visualize the differences between in vitro models, the relative expression of selected lncRNAs was compared with their levels in corresponding tumors and exhibited as fold change in relation to the parental GBs (Fig. 3b).

Almost all the analyzed lncRNAs showed significant changes in expression between initial tumor and the cultured cells: at least a tenfold change in lncRNA expression was observed between the in vitro and in vivo cultures for at last two tumors (*p* < 0.05). The highest discrepancies in lncRNA expression were presented by the G108-derived culture. The greatest change in expression of an individual lncRNA was observed for XIST in the case of G114 tumor; however, its level in the parental tumor was extremely low in comparison to HB (control).

Although the in vitro lncRNA expression profile seems to be more stable, differences were also noticed between particular models (*p* < 0.05). The most significant discrepancies were observed for H19 and MIR210HG, which demonstrated at least a tenfold change between particular models for the G108, G114 and G116 tumors. Minimum fivefold changes were detected for XIST, TUSC7, RP11-838N2.4 (for at least two analyzed tumors) and CASC2, GLIDR and DLX6-AS1 (for one analyzed tumors). MALAT1 and SOX2OT presented the most stable expression in vitro.

### Potential Influence of In Vitro Environment on Tumor Resistance Network via lncRNA-mRNA Interactions

As TMZ is a standard in GB treatment, the study examined a number lncRNAs known to influence TMZ resistance of glioblastoma cells. Most of the listed lncRNAs were previously recognized as competing endogenous RNAs (ceRNAs), which acted as “sponges” for the miRNAs regulating the expression of their target genes. Previous studies suggest that the effect of changes in target expression were detectable both at the mRNA and protein level (Table 3).

**Table 3:** Summary of interconnections of lncRNAs and their downstream target genes involved in drug resistance network

lncRNA	Relation to TMZ resistance	Target gene	Mechanism of lncRNA action
MALAT 1	Upregulated in TMZ-resistant GB and GB cell lines	TYMS	MALAT1 can promote GB chemoresistance and influence cell proliferation through suppressing miR-203 and promote TYMS (thymidylate synthase) expression (Chen et al. 2017)
		MDR1; MRP5; LRP1; ZEB1	MALAT1 downregulation reduces drug resistance through inhibiting the expression of MDR1, MRP5, LRP1 and ZEB1 genes; reduces the cell viability; influences EMT process (Li et al. 2017)
		GSK3β; MGMT	Acts as miRNA sponge to downregulated miR-101 and subsequently enhance the expression of GSK3β; miR-101 sensitized resistant GB cells to TMZ through downregulation of GSK3β; GSK3β inhibition increases MGMT promoter methylation resulted in downregulation of MGMT expression (Cai et al. 2018; Tian et al. 2016)
CASC2	Downregulated in patients showing no response to TMZ treatment and TMZ-resistant GB cells	mTOR	Acts as miRNA sponge to downregulated miR-193a-5p; involved in TMZ-induced autophagy by regulating mTOR (Jiang et al. 2018)
		PTEN	Upregulates PTEN through direct inhibiting miR-181a and plays an important role in glioma sensitivity to TMZ (Liao et al. 2017)
H19	Silencing H19 sensitizes GB cells to apoptosis;	MDR1; MRP5; ABCG2	Reduced level of H19 altered expression of drug resistance genes: MDR, MRP, and ABCG2 (Jiang et al. 2016)
	Highly expressed in TMZ-resistant GB cells	CDH1; VIM; ZEB1	Silencing of H19 suppressed epithelial-mesenchymal transition (EMT) by increasing the expression of epithelial marker E-cadherin and decreasing the expression of mesenchymal marker Vimentin and ZEB1; H19 decreased chemoresistance of glioma cells to TMZ by suppressing EMT via the inhibition of Wnt/β-Catenin pathway (Jia et al. 2018)
TUSC7	Negative correlation between expression level and TMZ resistance	MDR1	TUSC7 inhibited MDR1 expression by silencing miR-10a (Shang et al. 2018)
XIST	XIST knockdown can sensitize TMZ-resistant glioma cells to TMZ	SP1; MGMT	XIST/miR-29c coregulates SP1 and MGMT expression in TMZ-resistant GB cell lines and influence the chemoresistance of cells by modulating the MMR pathway (Du et al. 2017)
lncRNA RP11-838N2.4	Down regulated in TMZ-resistant GB cells;	EphA8	Reduces the expression of miR-10a and attenuates its inhibition of downstream targets EphA8 (Liu et al. 2016)

Since our results revealed differences in lncRNA expression between GB tumors and GB cells in vitro, the study also examined the expression pattern of selected genes recognized as targets of the analyzed lncRNAs (Table 3).

**Fig. 4:**
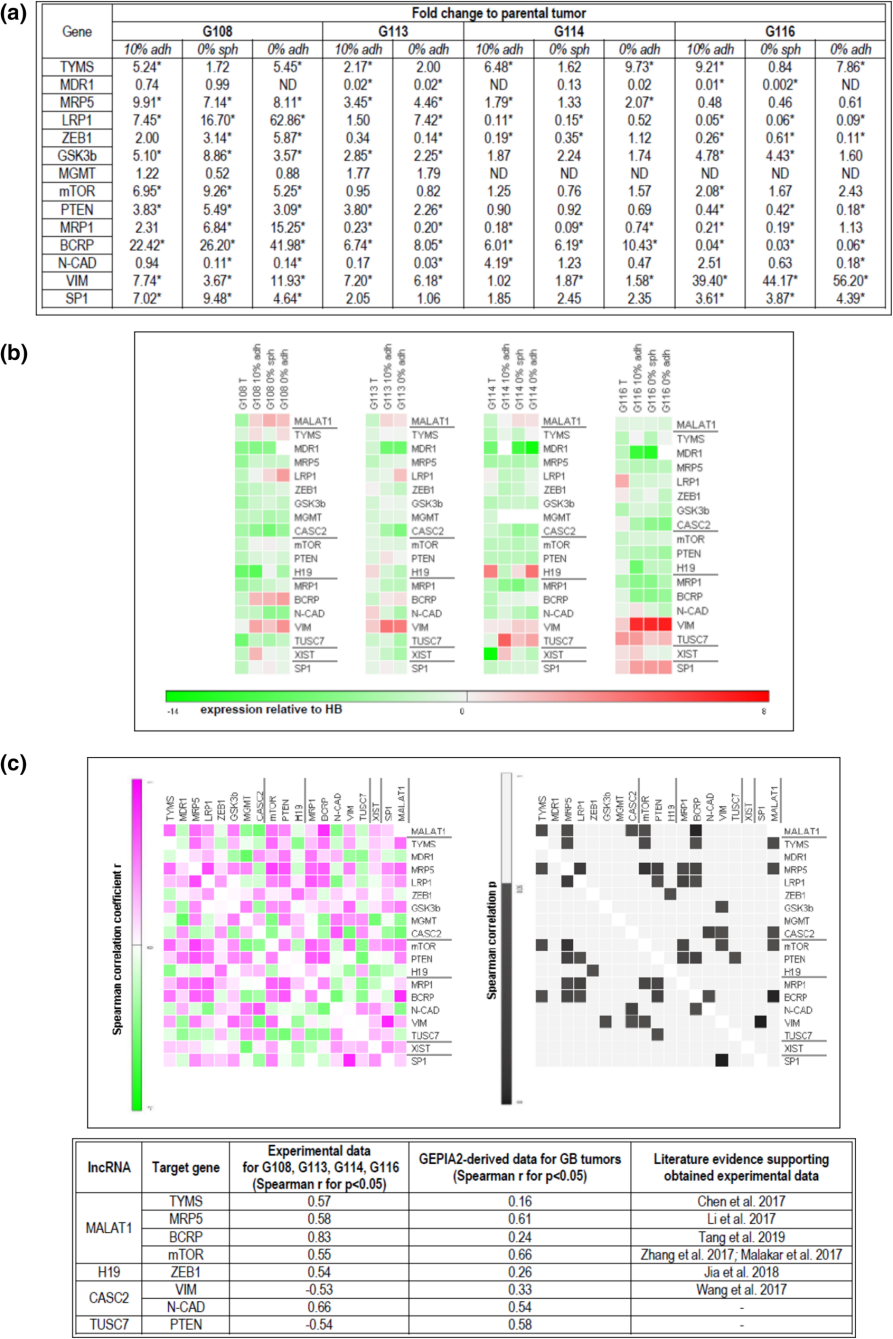
Analysis of potential interdependence of the studied lncRNAs and their downstream targets in vitro. The expression profile of genes recognized previously as targets for particular lncRNAs in GB-derived models in vitro, presented as fold change to parental tumor; differences in expression can be observed between in vivo and in vitro models, with the highest differences detected for *MDR1*, *MRP1*, *LRP1*, *BCRP*; (*) (for *p* < 0.05). (**a**) The results of parallel analysis performed for lncRNAs (underlined) and their target genes, visualized as heat maps generated from mean ΔΔCT values (relative to control—human brain) (**b**). The results of the co-expression analysis, presented as a correlation matrix. Potential relationships between examined lncRNAs and the selected genes listed in the table are given (based on Spearman’s correlation analysis, *p* < 0.05) (**c**)

The obtained results demonstrated significant differences (*p* < 0.05) in expression for all of the tested genes between parental tumors and the cells in vitro (detected for at least two per four tumors). However, the observed discrepancies were not as high as those detected in the case of lncRNAs: most of the tested genes demonstrated an approximate 5- to tenfold change of expression between in vitro and in vivo conditions. The greatest divergence was observed in the case of *MDR1*, *LRP1*, *BCRP* and *MRP1*, which presented at least a tenfold change when compared to parental tumors (Fig. 4a).

The in vitro models demonstrated more stable patterns of gene expression; however, *TYMS, LRP1, ZEB1, GSK3b, PTEN, VIM, MRP1* and *N-CAD* expression was found to differ between particular types of culture, showing about 2- to tenfold changes between models (*p* < 0.05). No significant differences were detected for the *MRP5, mTOR, BCRP, SP1* and *MGMT* genes (Fig. 4a).

To provide an overall view of examined lncRNAs and their target gene profiles, the results were expressed as relative to control HB (mean ΔΔCt values) and exhibited as heat maps (Fig. 4b).

Since a simple functional relationship had previously been evidenced for most of the examined lncRNAs and their downstream target genes, co-expression analysis of the lncRNAs and their targets was performed for all tested samples (Fig. 4c). Some of the detected associations confirmed previously published data regarding the functional relationships of certain lncRNAs in GB (MALAT1–TYMS; MALAT1–MRP5, H19–ZEB1; CASC2–VIM; CASC2–N-CAD); (Table 3, Fig. 4c). In addition, the following potential interconnections were identified: MALAT1–BCRP; MALAT1–mTOR; TUSC7–PTEN. To verify obtained outcomes, our co-expression results were compared to GEPIA2-derived correlation analysis basing on TCGA and GTEx data for GB tumors (Fig. 4c).

**Fig. 5:**
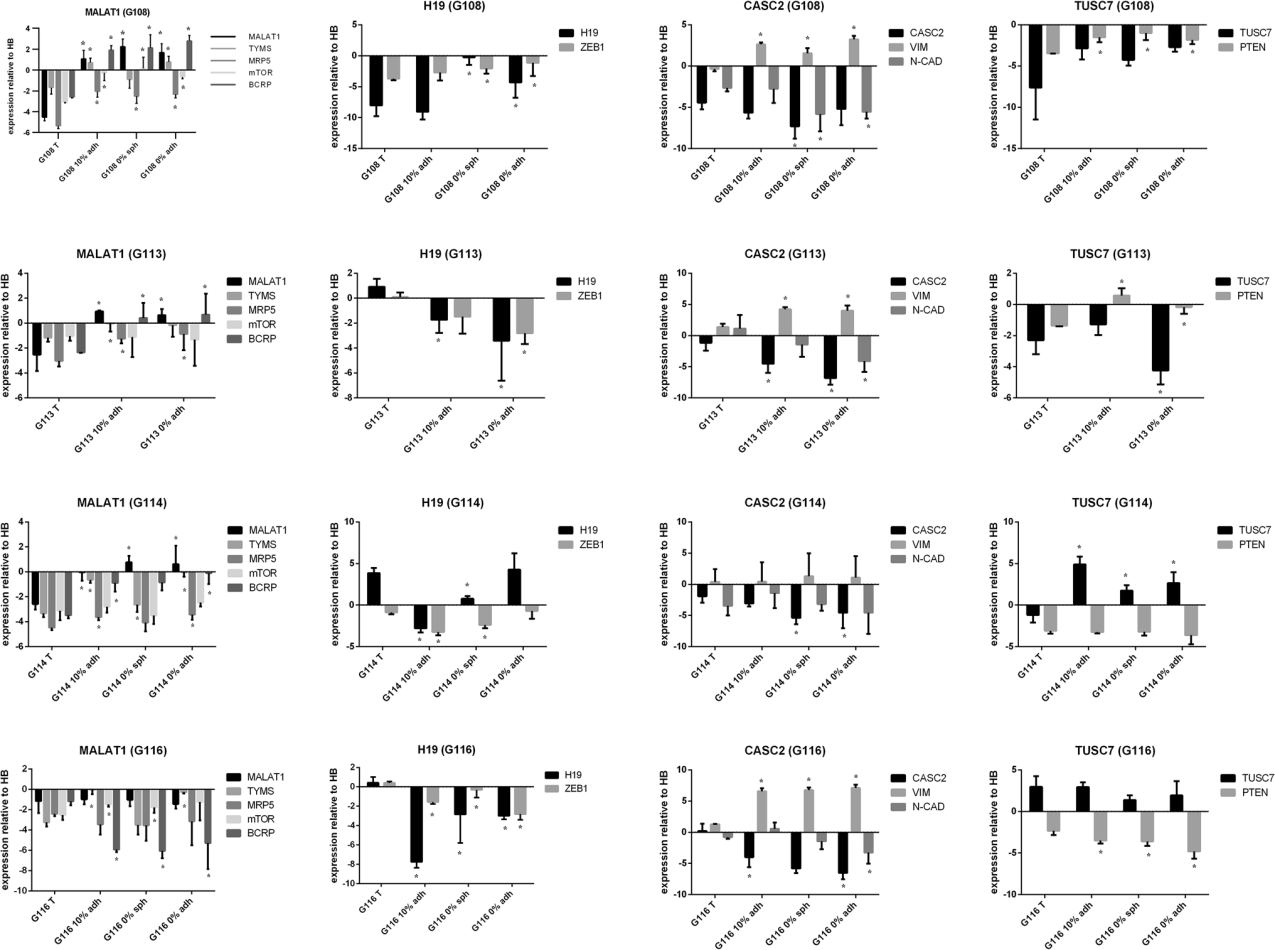
Expression pattern of the selected lncRNAs and their interrelated targets in GB initial tumors and in vitro models. The results were exhibited as expression relative to control HB (ΔΔCT values) and presented as means (± SD). Each bar chart is composed of results for single lncRNA and its potential target genes indicated on the basis of correlation analysis. Statistical significance (*) was labeled for comparison of initial tumors vs. particular in vitro models; *p* < 0.05. (The complete output of statistical analysis was Supplemented as Table S3)

The expression profiles of the selected lncRNAs and interrelated target genes are included in Fig. 5. The complete output of statistical analysis is provided as Table S3. Meticulous sample-by-sample analysis confirmed the most of indicated correlations, although the differences between particular tumors were observed. The more cohesive expression pattern of lncRNAs and matched targets was visible for tumors demonstrated higher changes in levels of examined lncRNAs (e.g. G108), in opposite to G116 showing lower variability in lncRNAs profile (e.g. MALAT1). However, expression pattern of TUSC7–PTEN pair seems to be partially inconsistent with indicated interrelation.

## Discussion

Of course, it is not surprising that an artificial in vitro microenvironment influences the molecular profile and phenotype of neoplastic cells, especially in case of such highly heterogeneous tumors as GB. Notwithstanding, tumor cell culture is still utilized as a basic tool for identifying new therapeutics. A number of studies have examined new methods of cell culture with the aim of improving representativeness compared to parental tumors and to maintain intra-tumor heterogeneity ex vivo (Balvers et al. 2017; Ledur et al. 2017; Robertson et al. 2019; Caragher et al. 2019; da Hora et al. 2019). Although, the influence of ex vivo factors on molecular profile of tumor-derived culture had been recognized previously, their effect on expression of lncRNAs in GB-derived cells has not been reported yet. What is important, expression of lncRNAs within tumor cell population undergoes dynamic changes, thus it could be more susceptible to extrinsic in vitro conditions (Hu et al. 2015). Consequently, the changes in lncRNAs profile may impact on their target gene expression and influence phenotype of tumor cells including their response to treatment.

The aim of our study was not to create an ideal in vitro glioblastoma model, but to analyze the profile of lncRNAs associated with tumor drug resistance in adherent culture with FBS, serum-free spheroid culture and serum-free adherent culture: these being the most popular experimental designs commonly used to investigate tumor responsiveness.

The artificial ex vivo environment has already been shown to influence tumor responsiveness, particularly the processes concerning cell death (Witusik-Perkowska et al. 2017), as well as the molecular basis of tumor resistance including the non-coding RNA (miRNA) (Witusik-Perkowska et al. 2019). Following on from these, our present findings indicate that the lncRNA profile related to tumor responsiveness is also influenced by ex vivo conditions.

The panel of examined lncRNAs were selected on the basis of a literature survey. The main criterion was their potential engagement in therapy response, with a special emphasis on TMZ treatment. The selected lncRNAs participate in GB therapy response via different pathways involving several target genes related to the following processes: multi-drug resistance (*MDR1*, *MRP5*, *MRP1*, *BCRP*), EMT (*N-CAD*, *VIM*, *ZEB1*), cell proliferation and viability (*TYMS*), autophagy (*mTOR*) and DNA repair (*GSK3β*; *MGMT*) (Table 3). All of the tested lncRNAs demonstrated different levels of expression between the in vitro cultures and the parental tumors. In addition, divergences in expression were observed between different in vitro models for some lncRNAs (Fig. 3).

Since most of the analyzed lncRNAs act as a “sponge” for specific miRNA, thus modulating their regulatory effect on mRNAs, we propose that changes in their levels in turn influence the expression of the target genes listed in Table 3*(*Paraskevopoulou and Hatzigeorgiou 2016; Long and Li 2019)*.* As expected, the genes recognized as targets of the examined lncRNAs demonstrated differential expression in vivo and in vitro; however, it was not possible to confirm that these disturbances resulted directly from changes in target-matched lncRNA levels. The highest discrepancies were detected for genes related to the phenomenon of multi-drug resistance (*MDR1, LRP1, BCRP* and *MRP1*). Functional studies in vitro have typically indicated simple relationships between an individual examined lncRNA and its targets (Table 3). The overall co-expression analysis allowed the identification of a few relationships between examined particles (Fig. 3), some of which (MALAT1–TYMS; MALAT1– MRP5, H19–ZEB1, CASC2–VIM; CASC2–N-CAD) have been reported previously (Chen et al. 2017; Li et al. 2017; Jia et al. 2018; Wang et al. 2020), while other potential interconnections are reported for the first time for GB: MALAT1–BCRP; MALAT1–mTOR; TUSC7–PTEN. Although further research is needed to examine the potential functional relationships of these particles, some support has been found in other studies. The most of our correlation results has been supported by experimental evidence based on gain and loss-function experiments performed previously for different tumor cells including glioblastoma (Fig. 4c).

MALAT1 is one of the best recognized lncRNAs involved in tumorigenesis and therapy resistance (Zhao et al. 2018; Chen et al. 2019). Chen et al. (2017) demonstrated that MALAT1 can induce chemoresistance to TMZ in GB cells through suppressing miR-203 and promoting thymidylate synthase (TYMS). The study by Li et al. confirmed interaction of MALAT1 and MDR genes (MDR1, MRP5, and LRP1) resulting in changes in TMZ responsiveness and ZEB1 expression (Li et al. 2017). Also the results by Tang et al. confirmed functional interrelation of MALAT1 with MDR1, MRP1 and BCRP in cancer cells (Tang et al. 2019a, b). PI3K/AKT/mTOR pathway, involved in a variety of cellular functions, is known to contribute in oncogenesis and cancer progression influencing cell cycle, metabolism, migration and cell death (Crespo Pomar and Arcaro 2016). Additionally, the activation of PI3K/Akt/mTOR pathway leads to the development of drug resistance thereby changing the effect of TMZ therapy (Li et al. 2016). PI3K/Akt/mTOR axis can be influenced by MALAT1 via interaction with the different miRNAs (miR-206; miR-146a) (Tang et al. 2018; Peng et al. 2020).

Tumor drug responsiveness is a complex phenomenon including EMT as a process involved in chemoresistance. Jia et al. demonstrated that silencing of lncRNA-H19 decreases chemoresistance of GB cells to TMZ by suppressing EMT via the Wnt/β-Catenin pathway. They showed that the expression of mesenchymal markers Vimentin and ZEB1 was downregulated by H19 shRNA (Jia et al. 2018).

EMT can be influenced by CASC2 via miR-18a suppressing also GB cell growth and metastasis. Wang et al. reported that overexpression of CASC2 resulted in downregulation of N-cadherin and Vimentin in GB cell lines, accompanied with tumor growth inhibition in vivo; whereas, silencing of CASC2 exerted the opposite effect (Wang et al. 2020). Molecular interrelation between CASC2 and Vimentin expression in tumor cells was also evidenced by Tu group (Wang et al. 2017).

Previous reports confirm the existence of functional connections between pairs of particles identified in other tumor types, or suggest a possible interaction between them during certain cellular processes (Tang et al. 2019a, b; Zhang et al. 2017; Malakar et al. 2017; Wang et al. 2017). GEPIA2-derived analysis confirmed our results for the majority of detected interconnections; however, the divergences were noticed for CASC2–VIM and TUSC7–PTEN pairs (table in Fig. 4c).

Our results suggest that expression of some target genes can be modulated by ex vivo factors via change of adequate lncRNA levels. However, the final phenotypic effect on drug resistance is difficult to predict, due to the complex nature of the lncRNA-miRNA-mRNA network and the interdependent cellular processes involved in drug resistance. The network is composed of particles engaged in a wide spectrum of cellular processes related to tumor drug sensitivity, including multi-drug resistance, EMT, autophagy, proliferation, viability and DNA repair.

The molecular and phenotypic intra-heterogeneity of GB presents a considerable challenge for ex vivo cell culture. All the more so because GB heterogeneity appears to be a major cause of tumor drug resistance (Cai and Sughrue 2017; Rybinski and Yun 2016; Parker et al. 2016; Akgül et al. 2019). Molecular intra-heterogeneity was first identified at DNA level, revealing a “patchwork” of cells carrying different genetic hallmarks of GB within a single tumor; however, it was later observed at transcriptional levels, including that of non-coding RNA (Kumar et al. 2014; Patel et al. 2014). The scale of intra-tumor heterogeneity at the lncRNA level has been described recently in two bioinformatic analyses of available datasets (Lv et al. 2016; Hu et al. 2015). Hu et al. (2015) emphasize the dynamics of changes in lncRNA expression identified across single cells in glioblastoma, and suggest that tumor cells employ the plasticity of lncRNA expression to adapt to microenvironmental conditions, leading to different cell fates. This is confirmed by Lv et al (2016), who also note that a studied tumors demonstrated higher transcriptional diversity than the established GB cell lines.

The heterogeneity of the GB cells may be further exacerbated by extrinsic environmental factors in vitro. These not only influence the expression profile of genes at the single cell level, but also induce the selection of clones with a specific expression pattern within a cell population.

Although our present findings, obtained with the use of three types of in vitro models derived from four tumors, do not reveal a fully cohesive expression pattern of analyzed lncRNAs dependent on applied ex vivo conditions, they do nevertheless indicate that some of analyzed tumors seem to be more susceptible to extrinsic factors, presenting considerable divergence between the lncRNA profiles of the tumor in vivo and in vitro models (e.g. G108). Hence, they demonstrate unequivocally that the artificial in vitro microenvironment changes the profile of lncRNAs related to tumor drug resistance; however, the final effect of this external influence may be contingent on changes in induced expression changes and clonal selection of cells. Using tumor primary culture as experimental models creates a chance to study tumor response including heterogeneity of particular cases (Ye et al. 2020). Such an approach could be useful in testing potential drugs targeted to specific molecular profile of individual tumors. On the other hand, it enables comparing response to standard drug of the molecularly/phenotypically different tumor cases. So, usefulness of particular GB-derived cultures as the potential experimental models should be evaluate in context of their molecular profile and phenotypic characteristics.

According to study by Meyer et al. naïve patient tumors include TMZ–resistant clones, thus propagation of GB-derived primary culture can be a way to reveal tumor resistance preexisting at a clonal level (Meyer et al. 2015). On the other hand, using alternative culture conditions may promote the cells representing different phenotypes and contribute to unveil tumor resistance potential latent by intra-tumor heterogeneity and plasticity (Witusik-Perkowska et al. 2017). Although, spheroids are recognized as advantageous ex vivo model, some GB tumors are not able to create it (Günther et al. 2008; Binder et al. 2016). Since our experience confirms this observation, we have developed an alternative serum- free in vitro model based on vitronectin-mimicking surface (Witusik-Perkowska et al. 2017, 2019). The hierarchical clustering analysis demonstrated the similarity of lncRNA profile in GB cells cultured as spheroids and monolayer in serum-free conditions. The current results for lncRNAs are cohesive with our previous findings for miRNA pattern and the study exploring the way of response to TMZ in GB cells cultured as different experimental models (Witusik-Perkowska et al. 2017, 2019).

Despite the fact, that serum-supplemented culture seems to be less valuable tool, it turned out that a subset of GB tumors are able to grow in vitro exclusively in serum presence (Maturi et al. 2020). Consistently to this report, our previous studies had also demonstrated presence of stemness markers (SOX2 and nestin) in GB cells cultured in serum presence (Witusik-Perkowska et al. 2017).

In the light of mentioned findings supported by our experience, selection of a single in vitro model limits the field of investigation eliminating tumors unable to grow in given conditions. Moreover, the changes in tumor characteristics induced by ex vivo factors should not be recognized hastily as useless artifacts, but this phenomenon may be also considered as manifestation of tumor resistance potential.

The heterogeneity, and plasticity, of tumor cells, regarded as a key challenge to overcoming tumor resistance in vivo, is also extremely difficult to accommodate when designing in vitro protocols (Rybinski and Yun 2016; Akgül et al. 2019). Perhaps, to investigate tumor drug resistance, it would be possible to monitor the changes in molecular profile and phenotype of GB cells ex vivo, and even benefit from the features unveiled by neoplastic cells influenced by extrinsic factors? Such an approach may be an alternative to the desperate efforts to maintain the original heterogeneity of GB in vitro, especially when considering the dynamically changing profile of non-coding RNA.

## Electronic supplementary material

Below is the link to the electronic supplementary material.

Electronic supplementary material 1 (DOCX 11 kb)

Electronic supplementary material 2 (XLSX 15 kb)

Electronic supplementary material 3 (XLSX 17 kb)

## Data Availability

All data generated or analyzed during this study are included in this published article and its supplementary information files.
