# Supplementary material for: If Artificial In Vitro Microenvironment Can Influence Tumor Drug Resistance Network via Modulation of lncRNA Expression?—Comparative Analysis of Glioblastoma-Derived Cell Culture Models and Initial Tumors In Vivo
Source: Cell Mol Neurobiol. 2020 Nov 27;42(4):1005–20. doi: 10.1007/s10571-020-00991-3 (PMC8942942; doi:10.1007/s10571-020-00991-3)
Supplement: Supplementary file 1 — Electronic supplementary material 1 (DOCX 11 kb) [file 10571_2020_991_MOESM1_ESM.docx]

Table S1

| Gene | Primer sequence |
| --- | --- |
| TYMS | F: CTGCCAGCTGTACCAGAGAT  R: ATGTGCATCTCCCAAAGTGT |
| MRP5 | F: CACCATCCACGCCTACAATAAA  R: CAC CGC ATC GCA CAC GTA |
| LRP1 | F: CAGCTGGCCATCGAGATCA  R: TCCAGTCTCTGAGCCTCATGC |
| ZEB1 | F: GCACCTGAAGAGGACCAGAG  R: TGCATCTGGTGTTCCATTTT |
| GSK3β | F: AGTGGTGAGAAGAAAGATGAGGTCTATC  R: TGACATAAATCACAGGGAGCGTC |
| mTOR | F: CACCCAAGCCTGGGACCTCTA  R: GGCTGGTTGGGGTCATATGTT |
| PTEN | F: CAAGATGATGTTTGAAACTATTCCAATG  R: CCTTTAGCTGGCAGACCACAA |
| VIM | F: TGTCCAAATCGATGTGGATGTTTC  R: TTGTACCATTCTTCTGCCTCCTG |
| SP1 | F: GCCGTTGGCTATAGCAAATGC  R: CCTCTCCACCTGCTGTGTCA |
| EphA8 | F: CCACCAGGGTATGTAAATATC  R: TGTGCTTTGAAGACCATTT |
